# Toward a holistic definition for Information Systems for Health in the age of digital interdependence

**DOI:** 10.26633/RPSP.2021.143

**Published:** 2021-11-01

**Authors:** Marcelo D’Agostino, Myrna Marti, Paula Otero, Daniel Doane, Ian Brooks, Sebastian Garcia Saiso, Jennifer Nelson, Luis Tejerina, Alexandre Bagolle, Felipe Medina Mejia, Daniel Luna, Walter H. Curioso, Viviane Lourenço, Victoria Malek, Gerardo de Cosio

**Affiliations:** 1 Pan American Health Organization Washington, D.C. United States of America Pan American Health Organization, Washington, D.C., United States of America; 2 International Consultant Argentina International Consultant, Argentina; 3 Hospital Italiano de Buenos Aires Buenos Aires Argentina Hospital Italiano de Buenos Aires, Buenos Aires, Argentina; 4 International Consultant Canada International Consultant, Canada; 5 University of Illinois Champaign United States of America University of Illinois, Champaign, Ill., United States of America; 6 Inter-American Development Bank Washington, D.C. United States of America Inter-American Development Bank, Washington, D.C., United States of America; 7 International Consultant Colombia International Consultant, Colombia; 8 Universidad Continental Lima Peru Universidad Continental, Lima, Peru; 9 International Consultant United States of America International Consultant, United States of America

**Keywords:** Health information systems, public health, health policy, eHealth policies, Sistemas de información en salud, salud pública, política de salud, políticas de eSalud, Sistemas de informação em saúde, saúde pública, política de saúde, políticas de eSaúde

## Abstract

The article’s main objective is to propose a new definition for Information Systems for Health, which is characterized by the identification and involvement of all the parts of a complex and interconnected process for data collection and decision-making in public health in the information society. The development of the concept was through a seven-step process including document analysis, on-site and virtual sessions for experts, and an online survey of broader health professionals. This new definition seeks to provide a holistic view, process, and approach for managing interoperable applications and databases that ethically considers open and free access to structured and unstructured data from different sectors, strategic information, and information and communication technology (ICT) tools for decision-making for the benefit of public health. It also supports the monitoring of the Sustainable Development Goals and the implementation of universal access to health and universal health coverage as well as Health in All Policies as an approach to promote health-related policies across sectors. Information Systems for Health evolves from preconceptions of health information systems to an integrated and multistakeholder effort that ensures better care and better policy-making and decision-making.

## RESUMEN

El objetivo principal de este artículo es proponer una nueva definición de los sistemas de información para la salud, que se caracterizan por la identificación y la participación de todas las partes involucradas en un complejo proceso interconectado de recopilación de datos y toma de decisiones en el ámbito de la salud pública en la sociedad de la información. El concepto se desarrolló en un proceso de siete pasos que incluyó el análisis de documentos, sesiones presenciales y virtuales con expertos y una encuesta en línea a profesionales de la salud en general. Esta nueva definición procura ofrecer un criterio holístico, un proceso y un enfoque para la gestión de bases de datos y aplicaciones interoperables que considere desde un punto de vista ético el acceso abierto y gratuito a datos estructurados y no estructurados de diferentes sectores, información estratégica y herramientas de tecnologías de la información y de la comunicación (TIC) para la toma de decisiones en beneficio de la salud pública. También brinda apoyo al seguimiento de los Objetivos de Desarrollo Sostenible y la ejecución del acceso universal a la salud y la cobertura universal de salud, así como la salud en todas las políticas como iniciativa para promover políticas relacionadas con la salud en todos los sectores. El concepto de sistemas de información para la salud implica una evolución desde lo que se consideraba anteriormente sistemas de información de salud hacia un esfuerzo integrado por parte de varios interesados directos que garantiza una mejora en la atención, la formulación de políticas y la toma de decisiones.

## RESUMO

O principal objetivo deste artigo é propor uma nova definição para Sistemas de Informação em Saúde, que são caracterizados pela identificação e participação de todas as partes de um processo complexo e interconectado para a coleta de dados e tomada de decisão em saúde pública na sociedade da informação. O conceito foi desenvolvido por um processo de sete passos incluindo análise de documentos, sessões presenciais e virtuais com especialistas e uma pesquisa on-line com profissionais de saúde generalistas. A nova definição busca oferecer uma visão, um processo e uma abordagem holística para gerenciar aplicativos e bases de dados interoperáveis que consideram eticamente o acesso aberto e gratuito a dados estruturados e não estruturados de diferentes setores, informações estratégicas e ferramentas de tecnologia da informação e comunicação (TIC) para tomadas de decisão em prol da saúde pública. Também sustenta o monitoramento dos Objetivos de Desenvolvimento Sustentável e a implementação do acesso universal à saúde e da cobertura universal de saúde, assim como a Saúde em Todas as Políticas como uma abordagem para promover políticas relacionadas à saúde em vários setores. Os Sistemas de Informação em Saúde evoluíram de pré-conceitos dos sistemas de informação de saúde para um esforço integrado e com muitas partes interessadas, assegurando melhor cuidado, formulação de políticas e tomada de decisão.

Article 14 of the Pan American Sanitary Code,^1^ signed in Havana, Cuba, in November 1924, stated that, “Each of the signatory Governments agrees to put in operation at the earliest practicable date a system for the collection and tabulation of vital statistics.” In consonance, in 1954, countries of the Americas expressed their interest in health data collection as a key action for the benefit of public health and agreed to complete a four-year report on health conditions, preferably of a statistical nature (1).

Various theories of systems and different definitions were developed in the 20th century. Ludwig Von Bertalanffy, who in 1969 published his general systems theory (GST), asserted that the notion of system is as old as European philosophy (2). Mario Bunge^2^ in 2004 defined a system as an entity composed of four basic but interrelated elements: composition, environment, structure, and mechanism (CESM) (3).

There have also been different interpretations of the complexity of health information systems, like AbouZahr and Boerma (2005) who commented that the word “system” implies a connected whole or organized process. They stated that, in practice, most countries’ health information systems lack such cohesion, having developed in a piecemeal way, fashioned by administrative, economic, legal, or donor pressures, and are invariably highly complex (4).

In today’s information society, open and free access to quality, trusted, and timely health-related data and information is one of the critical success factors of any effective health system. Information and communication technologies (ICTs) are critical for having faster and better information to help overcome some of the persistent obstacles of health information systems (5).

Health Metrics Network’s 2011 study showed that the basic foundations for a good health information system are inadequate in many low- and middle-income countries. Results also showed that countries which face the greatest health challenges generally have the weakest systems for gathering, managing, analyzing, and using information (6). Moreover, the COVID-19 pandemic has revealed the need to strengthen health information systems, especially in these countries (7).

Another challenge to face in this era is the preconceptions of many public health professionals about health information systems as isolated initiatives mainly focused on software development, vital statistics data, and/or electronic health records, not conceiving them as an integrated and multistakeholder effort to ensure better care and better policy-making and decision-making. These preconceptions could be related to the lack of awareness of the current context established by the information society and the data revolution, and therefore cannot provide a general framework for policy development and decision-making (8).

This article’s main objective is to introduce a more comprehensive new definition for Information Systems for Health, characterized by the identification and understanding of all the parts of a complex and interconnected processes for data collection and decision-making in public health in the information society. This definition focuses on establishing the needed level of interoperability so data can be processed, analyzed, and used for the benefit of a health-related system to support informed, evidence-based decision-making and policy-making.

## Health information in the context of digital interdependence

The age of digital interdependence, as stated by the United Nations Secretary-General, should guide actions to manage digital technologies to maximize benefits to society and minimize harms, with a far-sighted and wide-ranging view of the complex ways in which they interact with societal, environmental, ethical, legal, and economic systems. This innovative vision presents unprecedented opportunities, including the use of structured and unstructured data and information for improving public health. If used ethically, anyone, including governments and health-related and academic institutions, will benefit from this situation as never before in history (9).

Due to the uncontrolled increase in data and information on the Web, and considering the unlimited possibilities offered by ICTs, in August 2014, United Nations Secretary-General Ban Ki-moon asked an independent expert advisory group to make concrete recommendations to mobilize the data revolution as an essential component of sustainable development (10). The main recommendations are related to two global challenges: a) the challenge of invisibility (gaps in what we know from data, and when we find out); and b) the challenge of inequality (gaps between those with and without information, and what they need to know make their own decisions).

At the World Summit on the Information Society (WSIS), Geneva 2003 and Tunis 2005, countries adopted a common vision identifying the main principles and challenges toward a people-centered, inclusive, and development-oriented information society (11). In 2005, governments reaffirmed their commitment to the foundations of an information society and outlined the basis for implementation and follow-up in the Tunis Agenda for the Information Society (12). They also agreed on the creation of the Internet Governance Forum.^3^

On 20 September 2011, the Open Government Partnership was formally launched as a multilateral initiative that aims to secure concrete commitments from governments to promote transparency, empower citizens, fight corruption, and harness new technologies to strengthen governance (13). Since 2011, the World Health Organization (WHO) and the Pan American Health Organization (PAHO), the WHO Regional Office for the Americas, have been reinforcing the concept of Health in All Policies as an approach to promote health-related policies across sectors; therefore, it is critical to adapt the current concept of health information systems into Information Systems for Health as a mechanism that facilitates the collection and use of health-related data from different sectors and sources. This in turn will facilitate the process for monitoring the United Nations Sustainable Development Goals (SDGs), with particular emphasis on SDG 3 (Ensure healthy lives and promote well-being for all at all ages), and the PAHO/WHO Strategy for Universal Access to Health and Universal Health Coverage.

## MATERIALS AND METHODS

A review of the PAHO/WHO official strategies and resolutions was conducted in order to identify information that outlined the organizational policy and strategy toward health information systems. The following official documents of PAHO/WHO were taken as initial reference for group work: a) Collection and Use of Core Health Data (CD40/19), presented in 1997 by PAHO Member States on progress in the implementation of the initiative base/country profile data; b) Ten-year Evaluation of the Regional Core Data in Health Initiative (CE134/16), on accountability over 10 years of implementation of the initiative base/country data profiles; c) PAHO 48th Directing Council Resolution, Plan of Action for Strengthening of Vital and Health Statistics (CE142/15); and d) the Resolution Strategy and Plan of Action on eHealth, incorporating the use of ICTs in health.

**FIGURE 1.**
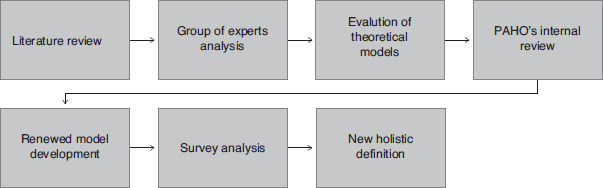
Road map for establishing the new holistic definition of Information Systems for Health

***Source:*** Prepared by the authors.

**TABLE 1.** Quantitative analysis of survey responses

Definition used in the survey: “A National Information System for Health is an integrated mechanism for the convergence of interconnected and interoperable systems, data, information, knowledge, processes, standards, people, and institutions, supported by the most cost-effective information and communication technologies that interact (or help) to generate, identify, collect, process, store, and make free and publicly available, quality data and strategic information for better policy and decision making processes in Public Health Systems.”							
	**Strongly agree**	**Agree**	**Neutral**	**Disagree**	**Strongly disagree**	**I need more information**	**Total responses**
**Level of agreement**	33.5% (156)	45.8% (213)	11.8% (55)	2.4% (11)	2.2% (10)	4.3% (20)	465

***Source:*** Prepared by the authors from the survey results.

A group of 10 experts in public health, information systems, information technologies, and health data management was formed in August 2015 to reflect on the modernization of the concept of health information systems in the context of the information society. They first met in a three-day activity oriented to a) review the current situation of the concept of health information systems, b) discuss the components that a renewed health information systems concept should include, and c) define a renewed health information systems model based on the general systems theory (GST) (2) to integrate all components of the system. During the sessions, technical institutional teams from PAHO/WHO were invited to provide input into the vision and expectations of a renewed model of health information systems. Different models and structures were evaluated, and the components, environment, structure, and mechanisms (CESM) model (3) designed was selected as being the most complete. Group work continued by electronic means such as email, videoconferencing, and online (Figure 1) until a renewed model was designed by PAHO/WHO.^4^

In total, 465 health professionals worldwide were consulted on the renewed model in the context of a broader online survey called Public Health in the Information Society. Their level of agreement was assessed, using a five-point Likert scale, with the question, “Please indicate your level of agreement with the following definition of ‘Information Systems for Health’: A National Information System for Health is an integrated mechanism for the convergence of interconnected and interoperable systems, data, information, knowledge, processes, standards, people, and institutions, supported by the most cost-effective information and communication technologies that interact (or help) to generate, identify, collect, process, store, and make free and publicly available, quality data and strategic information for better policy and decision-making processes in Public Health Systems.” All the answers and the 98 comments and suggestions were analyzed and incorporated to improve the model proposed. In general, 79.3% of the responders agreed (agree + strongly agree) with the definition used in the survey (Table 1).

While most people agreed with the definition used for the purpose of the survey (Table 1), some of them advised to keep it as simple as possible. There seemed to be an agreement that long and complex definitions tend to cause problems of misinterpretations instead of solving them.

## DISCUSSION

Direct, clear general concepts are more useful for readers to understand and for institutions to apply. Considering the feedback provided by the survey participants and the group of experts, the definition reads as follows: *Information systems for health is a holistic process and approach for managing interoperable applications and databases that ethically process structured and unstructured data from different sectors for the benefit of public health*.

**FIGURE 2.**
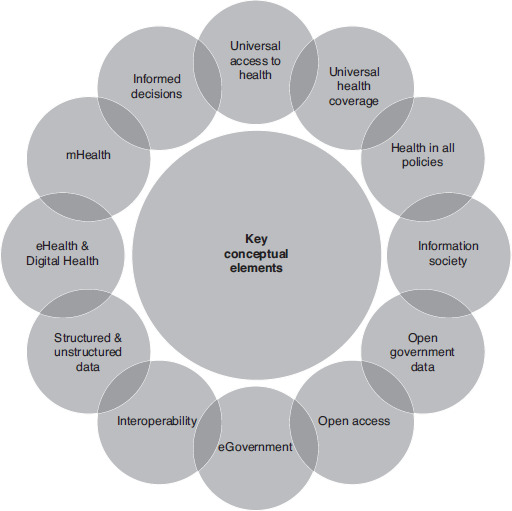
Key concepts for the new holistic definition for Information Systems for Health

***Source:*** Prepared by the authors.

Abraham Lincoln said, “Give me six hours to chop down a tree and I will spend the first four sharpening the axe.”^5^ The Merriam-Webster dictionary defines definition as “a statement expressing the essential nature of something.” It helps to have a common understanding and helps teams within institutions to organize collective thinking on what should be understood to achieve a successful project implementation. Therefore, having a common understanding of the definition of Information Systems for Health will contribute as the most critical factor to the development of the most appropriate project framework and its elements (14).

This new definition of Information Systems for Health seeks to provide a holistic view that considers open and free access to structured and unstructured data, strategic information, and ICT tools for decision-making and well-being. It also supports the implementation of universal access to health and universal health coverage as well as providing informed evidence to support Health in All Policies as an approach to promote health-related policies across sectors (15, 16).

Therefore, the new definition proposed by the authors embraces the key conceptual elements that are shown in Figure 2. In order to avoid having a circular definition or a simple theoretical one, the proposed definition for Information Systems for Health is intended to be operational and oriented to the specific purpose of strengthening a country’s capacity for health analysis, monitoring of the SDGs and predictions, and for supporting actions for universal access to health and universal health coverage (17). All the concepts were analyzed individually and collectively, including their points of convergence and their relation to public health.

## Disclaimer.

Authors hold sole responsibility for the views expressed in the manuscript, which may not necessarily reflect the opinion or policy of the *Revista Panamericana de Salud Pública/Pan American Journal of Public Health* and/or the Pan American Health Organization.

## Footnotes

1 The Pan American Sanitary Code was signed ad referendum by 18 countries of the Americas in the assembly hall of the former Academy of Medical, Physical, and Natural Sciences (now the Dr. Carlos J. Finlay Museum of Science History) on 14 November 1924 in Havana, Cuba, during the Seventh Pan American Sanitary Conference. *Source*: http://iris.paho.org/xmlui/handle/123456789/5668

2
https://www.mcgill.ca/philosophy/people/faculty/bunge

3 The Internet Governance Forum (IGF) is a multistakeholder forum for policy dialogue on issues of Internet governance.

4
https://www.paho.org/ish

5 *Source*: https://www.abrahamlincolnassociation.org/
